# Identification and Genetic Dissection of Resistance to Red Crown Rot Disease in a Diverse Soybean Germplasm Population

**DOI:** 10.3390/plants13070940

**Published:** 2024-03-24

**Authors:** Augustine Antwi-Boasiako, Shihao Jia, Jiale Liu, Na Guo, Changjun Chen, Benjamin Karikari, Jianying Feng, Tuanjie Zhao

**Affiliations:** 1 Key Laboratory of Biology and Genetics Improvement of Soybean, Ministry of Agriculture, Zhongshan Biological Breeding Laboratory (ZSBBL), National Innovation Platform for Soybean Breeding and Industry-Education Integration, State Key Laboratory of Crop Genetics & Germplasm Enhancement and Utilization, College of Agriculture, Nanjing Agricultural University, Nanjing 210095, China; bbbantwi@yahoo.com (A.A.-B.); chu1feng@163.com (S.J.); ljl15256082024@163.com (J.L.); guona@njau.edu.cn (N.G.); 2 Council for Scientific and Industrial Research-Crops Research Institute (CSIR-CRI), Fumesua, Kumasi P.O. Box 3785, Ghana; 3 College of Plant Protection, Nanjing Agricultural University, Nanjing 210095, China; changjun-chen@njau.edu.cn; 4 Department of Agricultural Biotechnology, Faculty of Agriculture, Food and Consumer Sciences, University for Development Studies, Tamale P.O. Box TL 1882, Ghana; benkarikari1@outlook.com; 5 Département de Phytologie, Université Laval, Québec, QC G1V 0A6, Canada

**Keywords:** soybean, resistance to red crown rot, germplasm evaluation, genome-wide association studies, candidate gene identification

## Abstract

Red crown rot (RCR) disease caused by *Calonectria ilicicola* negatively impacts soybean yield and quality. Unfortunately, the knowledge of the genetic architecture of RCR resistance in soybeans is limited. In this study, 299 diverse soybean accessions were used to explore their genetic diversity and resistance to RCR, and to mine for candidate genes via emergence rate (ER), survival rate (SR), and disease severity (DS) by a multi-locus random-SNP-effect mixed linear model of GWAS. All accessions had brown necrotic lesions on the primary root, with five genotypes identified as resistant. Nine single-nucleotide polymorphism (SNP) markers were detected to underlie RCR response (ER, SR, and DS). Two SNPs colocalized with at least two traits to form a haplotype block which possessed nine genes. Based on their annotation and the qRT-PCR, three genes, namely *Glyma.08G074600*, *Glyma.08G074700*, and *Glyma.12G043600,* are suggested to modulate soybean resistance to RCR. The findings from this study could serve as the foundation for breeding RCR-tolerant soybean varieties, and the candidate genes could be validated to deepen our understanding of soybean response to RCR.

## 1. Introduction

Soybeans [*Glycine max* (L.) Merrill] are grown worldwide mainly for their high values in oil and protein, yielding over 360 million metric tons in 2020 [1]. The need for soybean production continues increasing as it is essential in feeding humans, animals, and the industries for producing biofuel, ethanol, among others [2,3,4]. Unfortunately, several factors arising from biotic and abiotic stress are implicated in limiting the potential of soybeans to attain high yield and quality. Pathogens are well noted for causing significant economic losses in soybeans. For instance, in soybean production globally, bacteria, nematodes, viruses, and fungi are noted to cause 3, 5, 9, and 26 diseases, respectively [5].

The main causative agent of soybean red crown rot (RCR), *Calonectria ilicicola* Boedijn and Reitsma, is a soil-borne pathogen [6], becoming one of the most prevalent soybean diseases. It is alternatively called *Calonectria theae* Loos var. crotalariae Loos; *Calonectria crotalariae* (Loos) Bell and Sobers; and anamorph: *Cylindrocladium parasiticum* [7]. The RCR disease was first identified as *Cylindrocladium* black rot in groundnuts in 1965 [8] and then in soybeans in 1968 [9]. Until now, the disease has been present in Asia and Australia [10]. Specifically, in China, soybean RCR was first recorded in Jiangsu Province in 1997, causing 20% yield losses [11]. Reports on soybean RCR incidence were well documented in Taiwan (China) and the USA in 2019 and 2023 [12,13,14]. Similarly, in Japan, the soybean RCR incidence rate in the field ranges from 39 to 100% [15] and is ranked as the number one disease affecting soybean production [16]. Soybean *Calonectria ilicicola* (*C. ilicicola)* interactions threaten the soybean industry, affecting its quality and quantity [15,17]. Many states in America have recorded an estimated yield loss of about 25–30%, which is predicted to cause a 50–100% disease incidence in susceptible cultivars [12,16]. A reduction in yield loss of 50% is recorded under field conditions among susceptible cultivars [10,18]. The increases in the occurrences of soybean RCR are probably because the disease is seed-transmitted, because of its ability to spread via microsclerotia, and because of its low resistance level among genotypes [19,20,21].

To tackle the losses in soybean production instigated by RCR, it has been considered efficient, cost-effective, and environmentally sound to breed resistance. However, the few soybean cultivars evaluated against RCR exhibited small levels of resistance, though disparities in host resistance exist [21]. Hence, soybean germplasm is screened to detect their reactions towards RCR and to identify markers linked with RCR resistance. Moreover, genotypes conferring resistance to soybean RCR would facilitate breeding programs to improve RCR resistance.

Advancements in DNA markers facilitate the identification of quantitative trait loci (QTL) underlying partial resistance to diseases. Usually, linkage mapping (LM) or genome-wide association study (GWAS) strategies or both are utilized. GWASs based on natural population and high-density SNP markers have high levels of recombination events and shorter linkage disequilibrium (LD) blocks, resulting in enhanced resolution and accuracy for marker–phenotype associations [22,23]. Both methods are used to detect the genetic basis for resistance against soybean diseases such as white mold [24,25], bacterial leaf pustule [26], and *Phytophthora* root rot [27]. As of now, no research has been undertaken to pinpoint the QTL that contributes to the resistance of soybean RCR.

This study evaluated soybean RCR resistance levels in a panel of 299 diverse soybean germplasm from the National Center for Soybean Improvement, Nanjing Agricultural University, Nanjing, China. We applied multi-locus random-SNP-effect mixed linear model GWASs to identify soybean RCR resistance quantitative trait nucleotides (QTNs) and predict potential candidate genes near the peak and stable (SNPs). Findings from this would be useful for breeding programs aimed at marker-assisted selection (MAS) for resistance to RCR. Moreover, this study lays the foundation for exploring genes related to soybean RCR resistance.

## 2. Results

### 2.1. Response of Soybean Accessions to Red Crown Rot Strain

The 299 soybean accessions obtained worldwide were screened for their resistance to RCR under controlled conditions. The resistance response was determined by emergence rate (ER), survival rate (SR), and disease severity (DS) after the RCR strain inoculation. Soybeans affected by *C. ilicicola* show symptoms such as browning and softening of the root tips, stem coloration, yellowing and drooping of the leaves, reduced plant growth, and even death.

**Figure 1:**
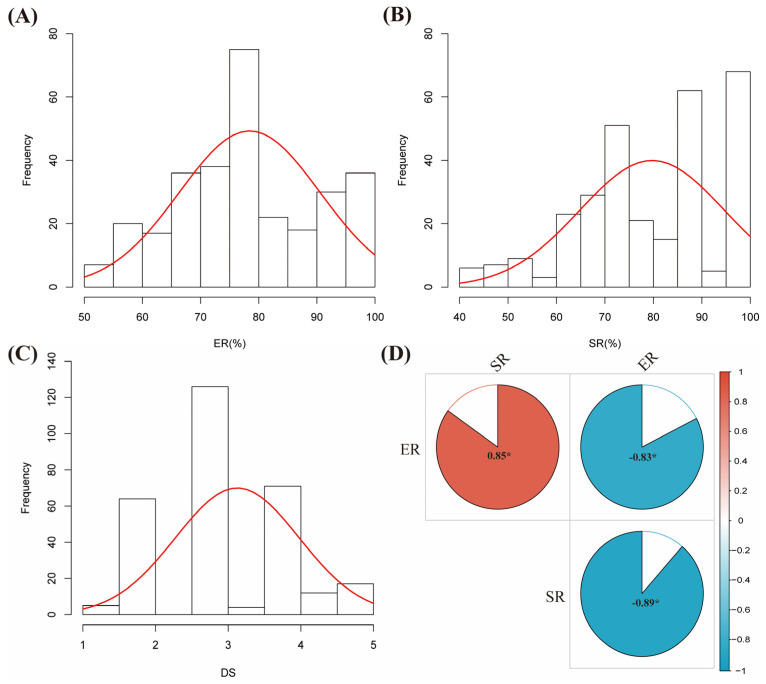
Phenotypic diversity of the 299 soybean accessions to RCR. (**A**) Emergence rate (ER, %), (**B**) survival rate (SR, %), (**C**) disease severity (DS), and (**D**) heatmap of Pearson correlation coefficients (r) among the ER, SR, and DS were significant at *p* < 0.05. The red curve on the frequency plots (**A**–**C**) represents a normal distribution line. * Significant at *p* < 0.05.

The results from ANOVA showed that the 299 accessions varied significantly (*p* < 0.05) in terms of ER, SR, and DS (Table S1). Except for DS, the ER and SR followed a continuous distribution (Figure 1A–C). This implies a broad range of diversity of resistance to RCR in the accessions used. Among the three parameters for soybean RCR, there is a significant (*p* < 0.05) correlation (Figure 1D). For instance, ER and SR positively correlated with a correlation coefficient (r) of 0.85, while DS negatively correlated with either ER (r = −0.83) or SR (r = −0.89). Our data indicated that DS directly affects the soybean’s emergence, survival rate, and, consequently, the crop yield.

### 2.2. Identification of Resistance to Soybean Red Crown Rot Strain

We observed high variability among the soybean panel for *C. ilicicola* resistance. The genotypes were screened using 0–5 scales for DS with six varied degrees of resistance in this work. None of the 299-soybean panel was identified as immune or highly resistant (DS = 0); however, five genotypes, namely PI 602496, PI 567731, PI 587880A, PI 424412, and PI 407196, were identified as resistant (DS = 1) with their greater ER and SR within 93 to 100% (Table S2). Nine of the materials (PI 547885, PI 567104B, PI 598124, PI 614833, PI 468967, PI 628889, PI 590931, PI 445681, and PI 567312) were identified as highly susceptible by the DS (DS = 5), and their ER and SR were less than 50% and 45%, respectively (Table S2). However, genotypes with DS of ≤2.5, ≤3.5, and >3.5 were classified as resistant, moderately resistant, and susceptible to *C. ilicicola*, respectively. In summary, out of the 299-soybean panel, 69, 131, and 99 genotypes were identified as resistant, moderately resistant, and susceptible, respectively (Table S2).

### 2.3. SNP Density and Distribution among the 20 Chromosomes of Soybean

**Figure 2:**
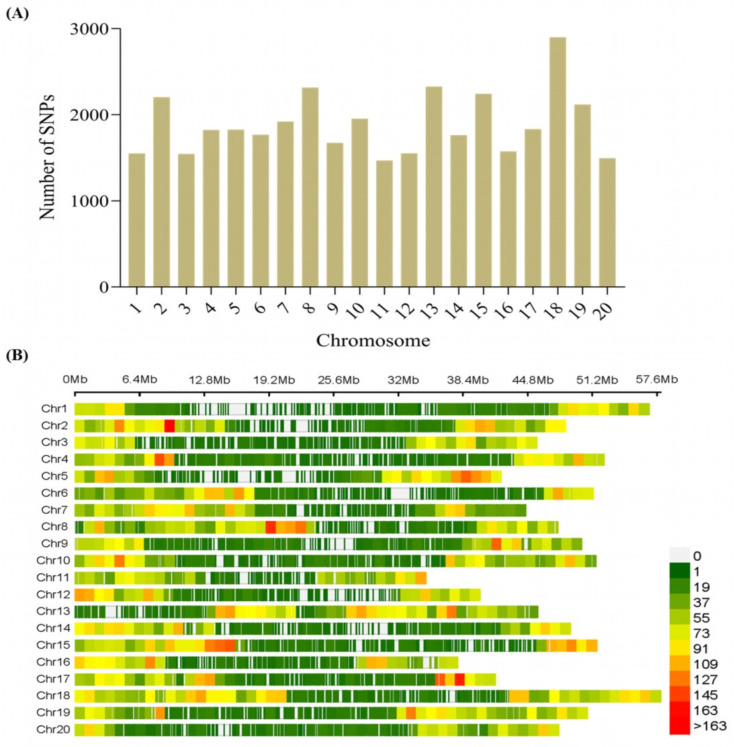
Distribution of high-quality SNPs across the chromosomes of soybean. (**A**) Number of SNPs per chromosome. (**B**) Number of SNPs within 1 Mb window size of each chromosome.

SNP density for mapping is documented to affect the power of detection of quantitative trait loci /nucleotides (QTL/Ns) [28]. The 299 accessions were genotyped with *Illumina Infinium* SoySNP50K BeadChip by [29]. We conducted quality control checks; thus, SNPs with a minor allele frequency (MAF) >0.05 and a missing rate of 5% were excluded for downstream analysis, leading to a total of 37,876 SNPs across the 20 chromosomes of soybean (Figure 2A). The maximum number of 2899 SNPs was located on chromosome 18 (Chr18), with the lowest (1470) on Chr11 (Figure 2A). In addition, the longest and shortest lengths were located on Chr18 and Chr11, respectively, with varied SNP densities (Figure 2B; Table S3).

### 2.4. Worldwide Soybean Germplasm, Its Population Stratification, Genetic Diversity, and Population Structures Based on Their Origin

**Figure 3:**
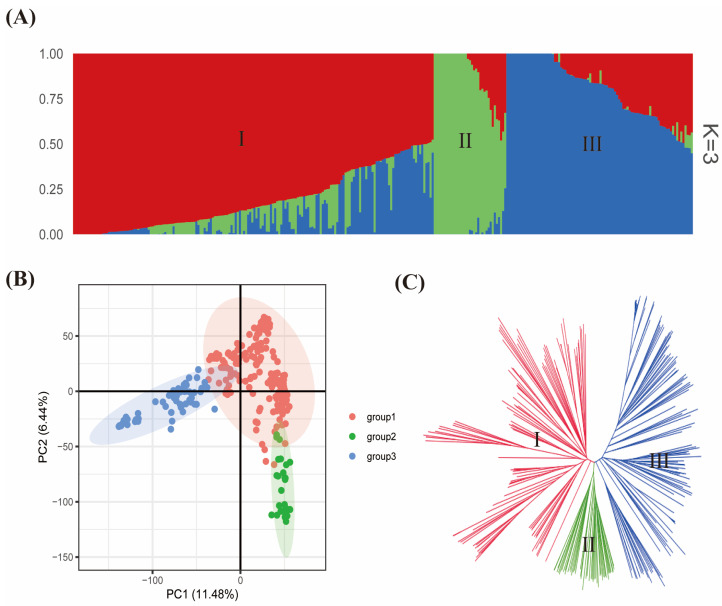
Population stratification of the 299 worldwide soybean collections used in this study based on the 37,876 SNPs across the 20 chromosomes. (**A**) Population structure obtained from ADMIXTURE software 1.3.0 with 2−10 runs. The I, II, and III represent subpopulations I, II, and III, respectively. Three colors (blue, green, and red) stand for three subpopulations. Each color represents one inferred ancestral population. A single individual is represented by each vertical column, and the percentage of each column’s colored segments reflects the individual’s presumed ancestral population among the 299 accessions. (**B**) Principal component analysis plot. The x- and y-axis represent PC1 and PC2, respectively, with their contribution to the total variability. (**C**) Neighbor-joining tree obtained from TASSEL software version 5.2 grouped the soybean collections into three clusters identified as I, II and III.

Population relatedness has been demonstrated to cause false positives in marker–trait association (MTA) mapping [30,31]. Consequently, it is required to further assess the extent of relatedness among the 299-soybean accession. The mapping population was optimally grouped into three subpopulations (i.e., I, II, and III) based on model-based analysis in ADMIXTURE software version 1.3.0 (Figure 3A). The groupings from ADMIXTURE software were largely similar to those obtained from principal component analysis (PCA) and neighbor-joining tree (Figure 3B,C). Also, the 299 accessions were grouped into three according to how the pairwise kinship coefficients are distributed. The subpopulation I/group I comprised *G. max* accessions from Asian countries. Meanwhile, the accessions in subpopulation II/group 2 consisted largely of *G. soja.* In contrast, the subpopulation III/group III largely included *G. max* accessions from the United States. The first two PCA axes accounted for 17.92% of variability among the 299 accessions in this study. The PCA pinpointed a high genetic diversity among the 299-mapping population.

The minor allele frequency (MAF), expected heterozygosity (*H_e_*), and polymorphic information content (PIC) on genetic diversity of the PIs based on their origin are shown in Table 1. Table 1 shows that MAF ranged from 0.14 for the Thia cultivar to 0.26 for the Russian germplasm. A similar pattern was observed for *H_e_*, varying from 0.19 to 0.35 and with PIC ranging from 0.16 to 0.27, thus showing a pattern of variation between nations that is comparable to MAF. In summary, a wide genetic diversity was observed from cultivars from China and Russia, whereas cultivars from Thailand and Japan exhibited narrower diversity.

**Figure 4:**
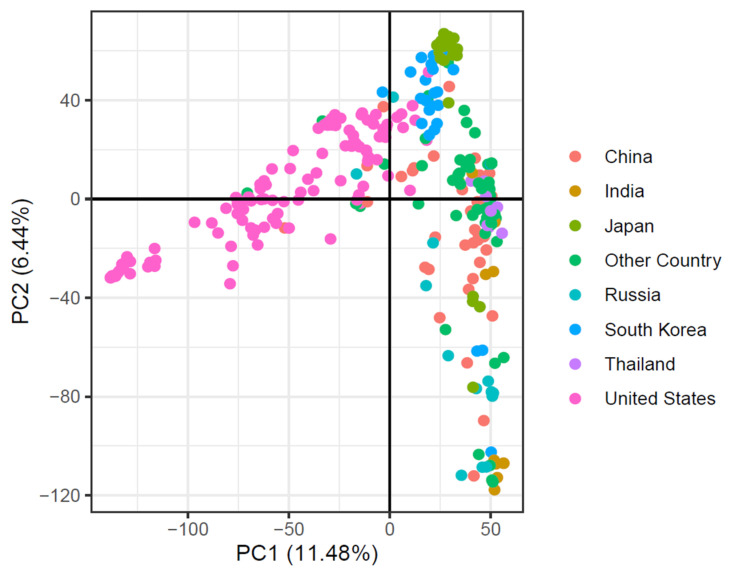
Principal component analysis of 299-soybean accession based on their country of origin.

Using SNP marker data, principal component analysis (PCA) was applied to evaluate the soybean accessions’ population composition based on their country of origin. The first two main components explained 17.92% of the overall genetic variability (Figure 4). The major population structure was revealed by the PCA based on the cultivar’s origin.

### 2.5. Marker–Trait Associations (MTAs)

**Figure 5:**
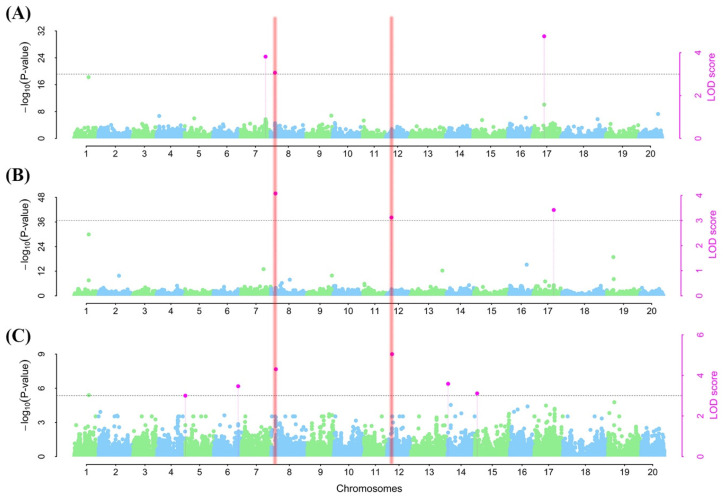
Manhattan plots for soybean red crown rot response via by emergence rate (**A**), survival rate (**B**), and disease severity (**C**) under a controlled environment in this study. The black dotted horizontal lines represent the threshold for significance at the logarithm of odd (LOD) = 3 and its corresponding −log (*p*-value). The blue and green dots fall below the threshold. The pink dots represent significantly linked SNPs to each of the studied indices. The vertical line in the (**A**–**C**) shows traits linked to a particular chromosome.

To detect SNPs with both major and minor effects for RCR tolerance/susceptibility, we employed the 3VmrMLM model from the 3VmrMLM package [32] and detected nine SNPs across eight chromosomes (i.e., Chr05, Chr06, Chr07, Chr08, Chr12, Chr14, Chr15, and Chr17) (Table 2). Three SNPs (ss715597632, ss715602602, and ss715625925) were associated with ER on Chr07, Chr08, and Chr17 with a phenotypic variance explained (PVE) of 5.44–7.64% (Table 2; Figure 5A). Of these SNPs, ss715597632 and ss715602602 contributed positively to ER, while ss715625925 reduced ER (Table 3).

On the other hand, one SNP (ss715602602) on Chr08 and two others, one each on Chr12 (ss715612097) and Chr17 (ss715627013), were associated with SR (Table 2; Figure 5B). The PVE values of these SNPs ranged from 5.29 to 7.56%. With the exception of ss715627013, the two other SNPs (ss715602602 and ss715612097) had a positive effect on SR (Table 2).

Furthermore, six SNPs with one each on Chr05 (ss715592629), Chr06 (ss715594897), Chr08 (ss715602602), Chr12 (ss715612097), Chr14 (ss715619777), and Chr15 (ss715621431) were linked to DS with PVEs of 5.09, 4.82, 6.80, 7.20, 3.58, and 3.11%, respectively (Table 2; Figure 5C). Of these SNPs, only ss715592629 (Chr05) and ss715619777 (Chr14) enhanced DS, while the remaining four SNPs reduced the DS (Table 2). Comparatively, two SNPs (ss715612097 and ss715627013 on Chr08 and Chr12, respectively) were associated with at least two of the indices used to assess RCR tolerance or susceptibility. These SNPs may be responsible for the high levels of correlation among the RCR indices (Table 2). These two SNPs were used to mine for potential candidate genes and their allelic effects.

### 2.6. Haplotype Analysis for the Identification of Superior Haplotypes and Candidate Genes Mining

**Figure 6:**
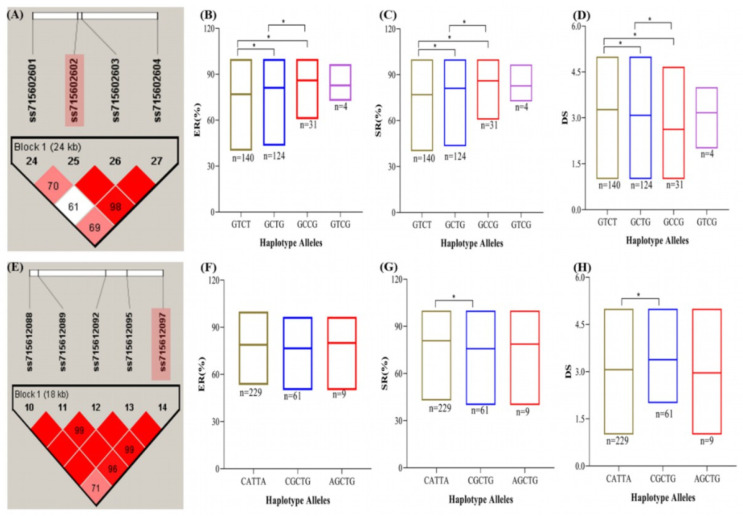
Haplo-phenotype analysis of stable single-nucleotide polymorphisms (SNPs) linked to at least two evaluated indices of soybean RCR response (shaded SNP). (**A**) Haplotype block around SNP ss715602602 on chromosome 8. (**B**) Phenotype (emergence rate, ER) grouping based on the four haplotype groups. (**C**) Phenotype (survival rate, SR) grouping based on the four haplotype groups. (**D**) Phenotype (disease severity, DS) grouping based on the four haplotype groups. (**E**) Haplotype block around SNP ss715612097 on chromosome 12. (**F**) Phenotype (emergence rate, ER) grouping based on the three haplotype groups. (**G**) Phenotype (survival rate, SR) grouping based on the three haplotype groups. (**H**) Phenotype (disease severity, DS) grouping based on the three haplotype groups. Means among haplotype groups were compared by a one-tailed *t*-test at 95% confidence level. * Significant at *p* < 0.05

To comprehend the phenotypic variances more fully among the 299 soybean accessions carrying a specific haplotype, we conducted haplo-phenotype analysis of the two stable SNPs (ss715612097 and ss715627013 on Chr08 and Chr12, respectively) (Table 4; Figure 6A–C). The haplotype around SNPs ss715612097 and ss715627013 spanned 24 and 18 kb, respectively (Figure 6A,E). Among the two stable SNPs, there were three to four different haplotype alleles underlying each block. For example, the SNP ss715612097 possessed four different alleles (GTCT, GCTG, GCCG, and GTCG), whereas SNP ss715627013 possessed three alleles (CATTA, CGCTG, and AGTG). The effects of haplotype alleles were tested on RCR resistance traits. On SNP ss715612097 (Chr08), the alleles GTCT, GCTG, and GCCG showed significant (*p* < 0.05) variation in ER, SR, and DS (Figure 6A–D). On the other hand, the haplotype block of ss715627013 (Chr12) divided the 299 soybean accessions into three groups (CATTA, CGCTG, and AGTG) (Figure 6E). Only CATTA and CGCTG haplotype groups showed significant variation (*p* < 0.05) in terms of SR and DS (Figure 6G,H).

In addition to identifying putative candidate genes around the two stable SNPs, we applied a haplotype block size up- and downstream of the SNPs to mine for putative candidate genes for soybean RCR. From this strategy, three probable genes (*Glyma.08G074500*, *Glyma.08G074600,* and *Glyma.08G074700*) were found in the haplotype block of ss715602602 (Chr08). *Glyma.08G074600* is annotated to be involved in plant disease responses in signaling mechanisms involved in the management of fungi (Table 3). Also, *Glyma.08G074700* is related to carbohydrate metabolic and xylan catabolic processes (Table 3). Moreover, six putative genes were detected around the SNP ss715612097 (Chr12) (Table 3). Out of these, *Glyma.12G043600* located 10.9 kb downstream encodes for protein tyrosine kinase which is involved in protein phosphorylation and could be a candidate for regulating the 299 accessions to RCR in soybean (Table 3). Therefore, around the two stable SNPs (ss715602602 (Chr08) and ss715627013 (Chr12), we suggest that *Glyma.08G074600*, *Glyma.08G074700,* and *Glyma.12G043600* may be involved in modulating soybean RCR response.

### 2.7. Analysis of Expression of Genes Associated with RCR Resistance

**Figure 7:**
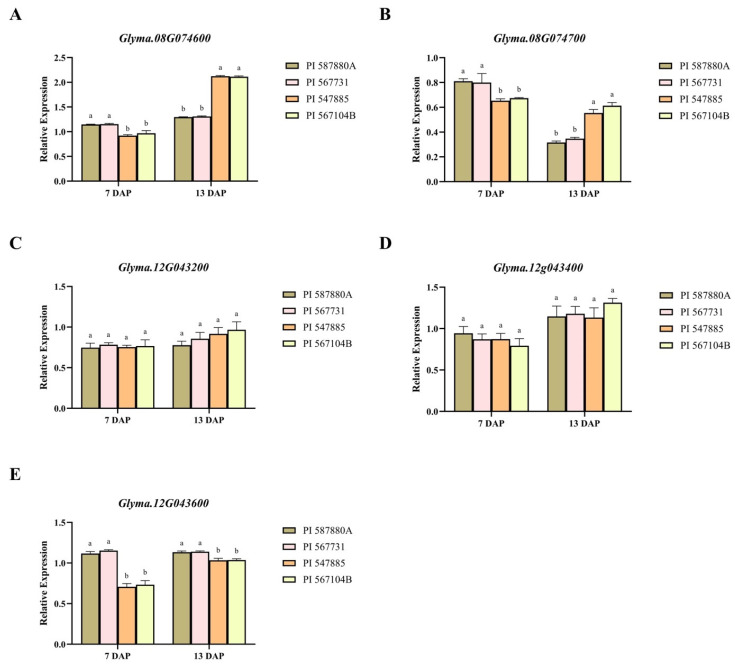
Expression patterns of five candidate genes (**A**) *Glyma.08G074600*, (**B**) *Glyma.08G074700*, (**C**) *Glyma.12G043200*, (**D**) *Glyma.12G043400*, and (**E**) *Glyma.12G043600*. Each putative gene’s mRNA concentrations in each candidate gene was analyzed in resistant soybean genotypes “PI 587880A and PI 567731” and susceptible genotypes “PI 547885 and PI 567104B”. A significant difference between means is marked by different letters (a and b) for each gene (*p* < 0.05). The different letters represent the significance differences.

We examined the pattern in transcript abundance of five selected genes (*Glyma.08G074600*, *Glyma.08G074700*, *Glyma.12G043200*, *Glyma.12G043400*, and *Glyma.12G043600*) in four soybean genotypes exhibiting a contrasting response under *C. ilicicola* inoculation. A similar gene expression was observed among the resistant genotypes (PI 587880A and PI 567731) and the susceptible genotypes (PI 547885 and PI 567104B) for genes *Glyma.08G074600*, *Glyma.08G074700*, and *Glyma.12G043600.* The gene expression level in resistant genotypes was up on the 7th day and decreased on the 13th day, and vice versa for the susceptible genotypes for *Glyma.08G074600* and *Glyma.08G074700* (Figure 7A,B). However, for *Glyma.12G043600,* the expression level was up on the 7th and 13th day in the resistant genotypes compared to the susceptible genotypes (Figure 7E).

## 3. Discussion

The effects of diseases on crops cannot be ignored in an attempt to feed the world’s expanding population while combating climate change. Every year, about 40% of yield losses are attributed to pathogens and pests [33], of which fungal diseases are known to cause 10–23% of losses [34], requiring much effort in controlling fungal diseases. Soybean crop is a key source of food for humans, making it valuable [35], and it needs to be protected from fungal diseases. Hence, host resistance in controlling soybean RCR is the most economical and sustainable approach. Soybean RCR incidence and severity are common in major soybean production areas globally [10,12,13,14].

### 3.1. Genetic Diversity among Soybean Germplasm

Understanding the global soybean collection’s genetic diversity is essential for regional breeding initiatives seeking to discover resistance genes. We found that the highest genetic diversity value of 0.31 was from the Chinese gene pool, but a decreased value was shown for Americans (0.28), which confirms the results of earlier research [36], with 0.41 recorded from sub-Saharan Africa [37]. Similarly, our results on the PIC value are comparable to findings on previously published values [36,37]. The comparatively high diversity of genes and PIC values recorded by others could be attributed to genetic materials included from countries such as Africa and also to the usage of simple sequence repeat markers, as the latter is reported for high diversity [38,39]. The reduced genetic diversity seen in countries like Thailand is probably due to robust selection criteria and smaller sample sizes than in other nations. A large soybean accession with different origins could offer an avenue for enhancing soybean breeding programs.

### 3.2. Evaluation and Identification of Resistance to Soybean Red Crown Rot Strain

Detecting novel sources of resistance in the soybean gene pool to key biotic stress, such as diseases, lays the foundation for improving productivity [5]. Several works on soybean improvement via enhancing resistance to diseases have been carried out globally [5,40,41], but limited efforts are geared towards soybean RCR disease. Evaluation of the soybean gene pool for disease resistance traits usually requires much labor and is, most of the time, cost-ineffective. The best alternative is genomic selection, leveraging marker data [42,43]. Accordingly, in-depth knowledge of disease resistance genetics is crucial to soybean yield development. In this circumstance, soybean genome sequences and the availability of numerous soybean SNP platforms offer crucial roles in supporting the development of the cultivar’s resistance to RCR.

Previous works on soybean’s reaction to RCR have detected a range of vulnerabilities. For instance, in 18 soybean cultivars evaluated in the field, none were observed with complete resistance [44]. Likewise, none of the 157 soybean genotypes consisting of cultivated and wild accessions were recorded as having complete resistance [45]. Also, Jiang and others [21] screened 213 soybean accessions and found none with high resistance. However, they found that wild soybean (*G. soja*) accessions exhibit high resistance compared to cultivated soybeans. The earlier outcomes are consistent with our finding; none of the 299-soybean panel (288 cultivated soybean or the 11 wild accessions) were recorded as highly resistant. Nevertheless, nine soybean accessions showed high resistance to *C. ilicicola* by DS and as resistant according to the ER and SR rating scale, and others were highly susceptible (Table S2). These variations could be utilized to develop recombinant inbred lines for QTL studies. These accessions lay the foundation for developing soybean cultivars resistant to *C. ilicicola*.

The identification of resistant material is the central phase towards managing RCR. Yet, understanding the complexity of inheritance governing resistance is crucial for successful breeding programs [46]. Evaluation of the panel of lines can be linked with diseases affecting soybeans, such as sudden death syndrome, bacterial leaf pustule, rust, and red leaf blotch disease [26,47,48,49]. However, there is no similar study conducted on red crown disease to date.

### 3.3. Marker–Trait Associations (MTAs), Haplotype Analysis, and Candidate Gene Mining

A GWAS was conducted using 299 PIs in a controlled environment of RCR disease and identified nine significant SNPs linked with either ER, SR, or DS. We used mrMLM since single-locus mapping models may not be able to detect all the QTNs [50]. Numerous studies confirm that resistance to root rot diseases in soybean is quantitative [51,52]. Several minor genes control quantitative resistance, contributing to partial resistance and reducing disease progression and its effects on plants [53]. Thus, there is a need for researchers to identify more QTL/QTNs linked with RCR disease resistance.

Additionally, the haplo-phenotype analysis revealed two stable significant SNPs for RCR disease traits. Specifically, the two SNPs (ss715602602 and ss715612097 on Chr08 and Chr12, respectively) were associated with at least two indices used to assess soybean reaction towards RCR. We identified four and three haplotype alleles on SNP ss715602602 and SNP ss715612097, respectively. The results revealed that the haplotype alleles GTCT, GCTG, and GCCG possessed by SNP ss715612097 showed significant variation in ER, SR, and DS, whereas CATTA and CGCTG possessed by SNP ss715627013 showed significant variation in SR and DS. The haplotype alleles that control the various genotypes’ RCR-resistance attributes allow breeders to alter soybean characteristics to suit their needs. Three putative genes were found in the haplotype block of ss715602602 (Chr08), and six putative genes were detected around the SNP ss715612097, making up nine (9) genes underlying RCR resistance. *Glyma.12G043600* belonged to the protein kinase family with a leucine-rich repeat (LRR) domain; *Glyma.08G074600* was an arginine/serine-rich protein that is engaged in signaling mechanisms involved in the management of fungi [54,55,56]. The *Glyma.08G074700* encoding the glycosyl hydrolase family, as well as protein tyrosine, is reported to promote resistance to fungus-causing leaf spot in tomatoes [57] and rice blast [58,59]. Thus, *Glyma.12G043600*, *Glyma.08G074600*, and *Glyma.08G074700* may be involved in modulating soybean RCR response based on the annotation in plant disease responses. The expression level of the predicted genes *Glyma.08G074600*, *Glyma.08G074700*, and *Glyma.12G043600* were appreciably upregulated in the resistant accession compared to the susceptible accession on the 7th day, which implies a possibility of its involvement in soybean’s reaction to *C. ilicicola* resistance. For instance, the *Glyma.08G074700* homolog in Arabidopsis, *AT5G64570*, encodes a secreted beta-d-xylosidase that enhances resistance to *Botrytis cinerea* [60] as well as boosts signaling related to systemic immunity in *Arabidopsis thaliana* [61].

The predicted candidate genes should be further validated to confirm their key roles in regulating *C. ilicicola* resistance. Also, there is limited understanding about the mechanisms underlying resistance to RCR among soybean genotypes. Previous works have been geared efforts towards developing efficient screening approaches called fresh-weight-based methods [62] and inoculum–soil mixtures [21], and towards identifying resistance sources [21,44,45]. Others have investigated the responses of tissue-specific expression to the *C. ilicicola* infection and the genes involved [63]. It has also been revealed that silicon enhances soybean’s resistance to RCR [64].

## 4. Materials and Methods

### 4.1. Seed Source, Planting Preparation, and Growth Conditions

The association mapping panel used consisted of 299 plant introductions (PIs), of which 288 are cultivated soybeans (*G. max* (L.) Merr.) and 11 are wild soybeans (*G. soja* sieb. & Zucc) from diverse countries across the globe (Table S2). The seeds were obtained from the National Center for Soybean Improvement, Nanjing Agricultural University, Nanjing, China.

### 4.2. Pathogen Culture, Inoculation, Planting, and Growth Conditions

The *C. ilicicola* strain Y62 was provided by the College of Plant Protection, Nanjing Agricultural University, Nanjing, China. The fungi were maintained on vegetable juice (V8) media plates (90 mm) at 26 °C for short-term use by subculturing and stored on a V8 slant at 5 °C for a more extended period (1 year) [65]. The mycelia of *C. ilicicola* Y62 strain was cultured on V8 media on Petri plates (9 cm) at 25 °C for 6 days. Pathogen inoculation was carried out following the protocol by [21]. Briefly, six mycelium plugs (~5 mm cubes) of V8 with actively growing *C. ilicicola* mycelia were placed in a 500 mL flask containing 200 g of wheat bran-vermiculite medium (wheat bran/vermiculite/water 1:1:3, *w*/*w*/*v*). It was then incubated at 26 °C for 14–21 days when the fungus had colonized the flask entirely. This formed the inoculum and was used to prepare inoculum–soil mixtures by mixing it with vermiculite soil to obtain a strength of 2% (*w*/*v*) and filling it into the plastic pot.

Seed coats of wild soybeans were scraped on its distal end towards the hilum to support water permeability. The media were composed of vermiculite and nutritive soil at 1:1 (*v*/*v*) and were autoclaved and filled in a plastic pot with drainage holes. The planting media were allowed to cool for two days at room temperature after which they were mixed with inoculum–soil mixtures to obtain a strength of 2% (*w*/*v*) and were filled into the plastic pot. Ten seeds were sown per pot, and the top was covered with a two-millimeter layer of the media with three biological replicates per line. Pots were placed in a container, and water was supplied to the pots via their drainage holes to ensure they were thoroughly wet in a greenhouse at 26 °C and 50% relative humidity. A supply of water to the container was performed when needed to maintain the soil wetness until the end of the assay. The pots were rotated every two days within the greenhouse to reduce any effects arising from the location of the plastic pots. The experiment was laid out as a completely randomized design with three replications. The means and the standard deviation were calculated using Microsoft Excel 2019.

### 4.3. Data Collection and Analysis

#### 4.3.1. Determination of Emergence Rate, Survival Rate, and Classification for Resistance to RCR

The soybean genotypes were scored for emergence rate (ER) on the 5th day after planting (DAP) and are expressed as the total number of seeds that emerged out of the total number of seeds planted expressed in percentage. The survival rate (SR) was taken on the 12th DAP and was calculated as the total number of plants alive out of the total number of plants that emerged expressed in percentage.

#### 4.3.2. Evaluation of Soybean for Resistance to *Calonectria ilicicola* and Statistical Analysis

Genotypes were scored for disease severity (DS) using the 0–5 scale on the 14th DAP (Table 4). Data collected on the DS, ER, and SR were subjected to analysis of variance (ANOVA) using GenStat software, version 12 (VSN International Ltd., UK). Pearson correlation analysis was performed among ER, SR and DS, and visualized in R with *Corrplot* package *p* < 0.05 [66].

### 4.4. Genotyping, Quality Control, and Population Structure Analysis

The SNP data were genotyped with *Illumina Infinium* SoySNP50K BeadChip [29]. SNP data were downloaded from the Soybase database (https://soybase.org/dlpages/#snp50k, accessed on 14 June 2023). A total of 42,506 SNPs were filtered using PLINK V1.9 [67], excluding SNPs with missing values exceeding 20% and a minor allele frequency (MAF) of less than 5% for quality control. This resulted in retaining 37,876 high-quality SNPs for subsequent analysis and investigation. Due to differences in the number of accessions used compared to the SoySNP50k dataset, we re-evaluated the population structure of the 299 soybean accessions using Admixture 1.3.0 (http://dalexander.github.io/admixture/download.html, accessed on 16 June 2023).

### 4.5. Genetic Diversity among the Soybean Accession Based on Their Origin and Statistical Accessed Analysis

The genetic materials were classified according to their origin (Table S2). Analysis of the principal component was executed in R package “popgen” [68] to generate data on genetic structure, variation, and diversity. Only countries with at least ten cultivars were considered in the computation of genetic diversity metrics by country of origin. Applying Nei’s genetic distances to serve as a basis, Ward’s minimum variance approach and the R package “stats” were used for grouping all the cultivars.

### 4.6. Multi-Locus Genome-Wide Association Analysis

Principal component analysis (PCA) and the kinship matrix were computed internally within R package GAPIT version 3. A threshold of −log_10_(*p*) ≥ 3 [69] was used to select 37,876 SNPs markers from 299 PIs significantly associated with the study traits (ER, SR, and DS). Using phenotypes of the three study traits, GWAS was implemented using the mrMLM package [70]. We conducted GWAS for all the study traits using the 3VmrMLM model from the 3VmrMLM package [32]. The default threshold LOD value of three was used for all the study traits in detecting significant QTNs. By aligning each significant SNP’s reference sequence to a soybean reference genome Wm82.a2.v1 from the SoyBase (http://www.soybase.org, accessed on 18 August 2023), the physical map placements of each SNP were found.

### 4.7. Haplotype Analysis and Candidate Gene Analysis

Haplotype analysis was carried out using Haploview software 4.2 [71]. The stable SNP marker blocks identified were used as the reference markers. To detect the possible candidate genes around the stable SNPs significantly associated with study trait, the haplotype block size was applied up- and downstream of the SNPs to mine for putative candidate genes for RCR resistance. Candidate genes were retrieved from the reference annotation of the soybean reference genome Wm82.a2.v1 from the SoyBase (http://www.soybase.org, accessed on 30 June 2023).

### 4.8. RNA Extraction and qRT-PCR

Five potential candidate genes around peak SNPs were selected for qRT-PCR to assess their transcript abundance under RCR conditions. We used two resistant (PI 587880A and PI 567731) and two susceptible (PI 547885 and PI 567104B) lines from the phenotypic screening. The planting preparation, growth conditions, pathogen culture, and inoculation are elaborated above. Root samples under RCR infection and control treatment were taken on the 7th and 13th DAPs with three biological and technical replicates. Total RNA was extracted from the roots and further synthesized into cDNA using Ultrapure RNA Kit (CWBIO, Taizhou, China) and HiScript II QRT SuperMix for qPCR (+gDNA wiper) (Vazyme, Nanjing, China), respectively. Primer 5 software was used to design the qPCR primer (Table S4) and the soybean actin (*Glyma.18G290800*) gene was used as the internal reference for standardization [72]. The ChamQSYBR qPCR master Mix Kit (Vazyme, Nanjing, China) was used for the qRT-PCR assay using the Light Cycler 480 system (Roche, Roche Diagnostic, Basel, Switzerland). The 2^−ΔΔCt^ method was used to calculate expressions [73]. Data were analyzed using the R package through the least significant difference (LSD) test at *p* < 0.05 and graphs were made using GraphPad Prism software 9.5.0.

## 5. Conclusions

We found five distinct soybean accessions (PI 602496, PI 567731, PI 587880A, PI 424412, and PI 407196) with high levels of partial resistance to *C. ilicicola*. Also, the current study presents the first report on marker–trait associations (MTAs) and stable SNPs for soybean RCR disease coupled with its genetic diversity based on cultivar origin. We employed the GWAS, haplotype analysis, and candidate gene mining to unravel the genetic architecture for soybean RCR resistance. We used the mrMLM model to detect nine significant SNPs and two stable SNPs (ss715612097 and ss715627013 on Chr08 and Chr12, respectively). Additionally, nine (9) genes underlying these two SNPs were identified, of which we speculate three of them to be prioritized as potential candidate genes. This study provides insights into the genomic regions of RCR traits. The MTAs identified could facilitate the breeding of new soybean varieties with resistance to RCR disease through the application of MAS after validation and testing in soybean germplasm. The candidate genes identified should be validated and employed for developing RCR-resistant soybeans. The studies could contribute to finding novel ways to develop soybeans against red crown disease. Our study critically analyzed soybean accessions and detected novel SNPs for soybean disease improvement programs.

## Figures and Tables

**Table 1:** Genetic diversity of accessions based on the origin.

Country	No. of Cultivars	MAF ^a^	*H_e_* ^b^	PIC ^c^
China	35	0.23	0.31	0.25
India	11	0.19	0.26	0.21
Japan	28	0.18	0.24	0.20
Russia	14	0.26	0.35	0.27
South Korea	26	0.20	0.27	0.22
Thailand	10	0.14	0.19	0.16
United States	115	0.22	0.28	0.23

^a^ minor allele frequency. ^b^ expected heterozygosity, ^c^ polymorphic information content.

**Table 2:** Nine SNPs detected to associate with parameters used to measure red crown rot among 299-soybean panel by mrMLM.

Trait ^a^	SNP ^b^	Chr ^c^	SNP pos ^d^	LOD ^e^	PVE (%) ^f^	QTN Effect ^g^	MAF ^h^	Genotype ^i^
ER	ss715597632	7	37631217	3.81	7.64	4.81	0.14	AA
	ss715602602	8	5709053	3.06	5.44	2.82	0.48	CC
	ss715625925	17	12730065	4.76	6.37	−8.11	0.50	TT
SR	ss715602602	8	5709053	4.08	6.43	3.88	0.48	CC
	ss715612097	12	3146531	3.12	5.29	4.22	0.22	AA
	ss715627013	17	36384272	3.42	7.56	−4.33	0.39	AA
DS	ss715592629	5	37228	3.00	5.09	0.22	0.28	AA
	ss715594897	6	48464349	3.47	4.82	−0.22	0.30	GG
	ss715602602	8	5709053	4.30	6.80	−0.23	0.48	CC
	ss715612097	12	3146531	5.05	7.10	−0.29	0.22	AA
	ss715619777	14	652974	3.58	4.68	0.22	0.25	AA
	ss715621431	15	2649998	3.11	4.31	−0.20	0.34	GG

^a^ Red crown rot emergency rate (ER), severity rate (SR), and disease severity (DS). ^b^ Single-nucleotide polymorphism (SNP); those with single and double underlines represent SNPs detected for two (SR and DS) and three (ER, SR, and DS) traits, respectively. ^c^ Chromosome (Chr). ^d^ SNP positions in base pair (Wm82 genome, version 2). ^e^ Logarithm of odd (LOD) with a threshold of 3. ^f^ Phenotypic variance explained (PVE). ^g^ Quantitative trait nucleotides’ effect; those with positive and negative effects represent increase and decrease in the traits, respectively. ^h^ Minor allele frequency (MAF) of the significant SNPs. ^i^ Significantly associated genotypes’ alleles.

**Table 3:** Putative genes within the two stable single-nucleotide polymorphism markers linked to at least two parameters used to access the 299 accessions to soybean red crown rot.

Chr_SNP_Position	Gene ID/Name ^a^	Position (bp)	Annotation Descriptions ^b^
Chr08_ ss715602602_ 5709053	*Glyma.08G074500*	5684546–5692295	BRI1-associated receptor kinase, protein phosphorylation, leucine-rich repeat
	*Glyma.08G074600*	5695514–5698741	Splicing factor, arginine/serine-rich, nucleic acid binding
	*Glyma.08G074700*	5707293–5712830	Carbohydrate metabolic process, xylan catabolic process
Chr12_ ss715612097_ 3146531	*Glyma.12G043100*	3128278–3132475	Carbohydrate metabolic process, glycosyl hydrolase
	*Glyma.12G043200*	3132968–3138908	Phenylalanyl-tRNA synthetase; tRNA aminoacylation; tRNA aminoacylation for protein translation
	*Glyma.12G043300*	3142970–3144547	Nucleic acid binding
	*Glyma.12G043400*	3143730–3147610	Erythronate-4-phosphate dehydrogenase
	*Glyma.12G043500*	3148801–3150720	BTB/POZ domain-containing protein
	*Glyma.12G043600*	3157432–3162480	Protein phosphorylation; protein tyrosine kinase; leucine-rich repeat

^a^ model gene retrieved on reference genome V2 from SoyBase (https://www.soybase.org/ accessed on 10 January 2024). ^b^ Gene ontology of the retrieved genes from SoyBase (https://www.soybase.org/ accessed on 10 January 2024).

**Table 4:** Disease resistance rating scale for DS.

Scale	Damage Degree	Resistance Degree
0	No visible sign of necrotic lesions on root	Immune
1	Only tap root exhibits small necrotic lesions without obvious changes in its form and shape	Resistant
2	Necrotic lesions spread to the crown and root system and seedling mortality less than 10%	Moderately Resistant
3	Roots show serious necrotic lesions with less than 50% loss by rot and seedling mortality of 10–20%	Moderately Susceptible
4	Roots show serious necrotic lesions with more than 50% root loss by rot and seedling mortality of 21–50%	Susceptible
5	Over 50% of root loss by rot with seedling mortality of more than 50%	Highly Susceptible

The determination of the resistance level among the germplasm was based on DS with supporting parameters of SR and ER. SR > 0.90 and ER > 0.85 were treated as a standard for identifying resistance germplasm. The SR assisted in determining the seedling’s mortality rate incorporated into the disease resistance rating scale. The DS uses a scale from 1 to 5 (Table 4). These were used to classify genotypes into different reactions based on RCR infection.

## Data Availability

Data are available within the article.

## Supplementary Materials

The following supporting information can be downloaded at https://www.mdpi.com/article/10.3390/plants13070940/s1. Table S1: Analysis of variance of three traits for RCR evaluation of the 299 worldwide soybean accessions. Table S2: A list of origin and phenotypic performances of three traits of 299 tested PI accessions. Table S3: Single-nucleotide polymorphism (SNP) density among the 299 worldwide soybean accessions used in this study. Table S4: List of primers used for the qPCR assay.

## Abbreviations

ANOVA	Analysis of variance
DAP	Day after planting
DS	Disease severity
ER	emergence rate
GWAS	Genome-wide association studies
MATs	Marker–trait associations
qRT-PCR	Real-time quantitative polymerase chain reaction
QTL	Quantitative trait loci
QTNs	Quantitative trait nucleotides
RCR	Red crown rot
SNP	Single-nucleotide polymorphism
SR	survival rate

## References

[1] Duan Z., Li Q., Wang H., He X., Zhang M. (2023). Genetic regulatory networks of soybean seed size, oil and protein contents. Front. Plant Sci..

[2] Sekhon J.K., Maurer D., Wang T., Jung S., Rosentrater K.A. (2018). Ethanol production by soy fiber treatment and simultaneous saccharification and co-fermentation in an integrated corn-soy biorefinery. Fermentation.

[3] Debnath D., Babu S.C. (2020). Prospects for sustainable intensification of soybean production in sub-Saharan Africa. Afr. J. Agric. Resour. Econ..

[4] Vedovatto F., Bonatto C., Bazoti S.F., Venturin B., Alves Jr S.L., Kunz A., Steinmetz R.L., Treichel H., Mazutti M.A., Zabot G.L. (2021). Production of biofuels from soybean straw and hull hydrolysates obtained by subcritical water hydrolysis. Bioresour. Technol..

[5] Lin F., Chhapekar S.S., Vieira C.C., Da Silva M.P., Rojas A., Lee D., Liu N., Pardo E.M., Lee Y.-C., Dong Z. (2022). Breeding for disease resistance in soybean: A global perspective. Theor. Appl. Genet..

[6] Crous P., Wingfield M., Alfenas A. (1993). *Cylindrocladium parasiticum* sp. nov., a new name for C. crotalariae. Mycol. Res..

[7] Crous P.W. (2002). Taxonomy and Pathology of Cylindrocladium (Calonectria) and Allied Genera.

[8] Bel D., Sobers E.K. (1966). A Peg, Pod, and Root Necrosis of Peanuts Caused By a Species of Calonectria. Phytopathology.

[9] Berggren G., Snow J., Sinclair J.B., Backman P.A. (1989). Red crown rot. Compendium of Soybean Disease.

[10] Hartman G., Rupe J., Sikora E., Domier L., Davis J., Steffey K. (2015). Compendium of Soybean Diseases and Pests.

[11] Wrather J., Anderson T., Arsyad D., Tan Y., Ploper L.D., Porta-Puglia A., Ram H., Yorinori J. (2001). Soybean disease loss estimates for the top ten soybean-producing counries in 1998. Can. J. Plant Pathol..

[12] Kleczewski N., Plewa D., Kangas C., Phillippi E., Kleczewski V. (2019). First report of red crown rot of soybeans caused by Calonectria ilicicola (anamorph: *Cylindrocladium parasiticum*) in Illinois. Plant Dis..

[13] Liu H., Shen Y., Chang H., Tseng M., Lin Y. (2020). First report of soybean red crown rot caused by Calonectria ilicicola in Taiwan. Plant Dis..

[14] Neves D.L., Mehl K.M., Bradley C.A. (2023). First report of red crown rot, caused by Calonectria ilicicola, and its effect on soybean in Kentucky. Plant Health Prog..

[15] Akamatsu H., Fujii N., Saito T., Sayama A., Matsuda H., Kato M., Kowada R., Yasuta Y., Igarashi Y., Komori H. (2020). Factors affecting red crown rot caused by Calonectria ilicicola in soybean cultivation. J. Gen. Plant Pathol..

[16] Sugimoto T., Kato M., Yoshida S., Matsumoto I., Kobayashi T., Kaga A., Hajika M., Yamamoto R., Watanabe K., Aino M. (2012). Pathogenic diversity of Phytophthora sojae and breeding strategies to develop Phytophthora-resistant soybeans. Breed. Sci..

[17] Roy K., McLean K., Lawrence G., Patel M., Moore W. (1989). First report of red crown rot on soybeans in Mississippi. Plant Dis..

[18] Berner D., Berggren G., Snow J., White E. (1988). Distribution and management of red crown rot of soybean in Louisiana. Appl. Agric. Res..

[19] Glenn D., Phipps P., Stipes R. (2003). Incidence and survival of *Cylindrocladium parasiticum* in peanut seed. Plant Dis..

[20] Randall-Schadel B., Bailey J., Beute M. (2001). Seed transmission of *Cylindrocladium parasiticum* in peanut. Plant Dis..

[21] Jiang C.-J., Sugano S., Ochi S., Kaga A., Ishimoto M. (2020). Evaluation of *Glycine max* and Glycine soja for resistance to Calonectria ilicicola. Agronomy.

[22] Li Y.-H., Reif J.C., Ma Y.-S., Hong H.-L., Liu Z.-X., Chang R.-Z., Qiu L.-J. (2015). Targeted association mapping demonstrating the complex molecular genetics of fatty acid formation in soybean. BMC Genom..

[23] Varshney R.K., Terauchi R., McCouch S.R. (2014). Harvesting the promising fruits of genomics: Applying genome sequencing technologies to crop breeding. PLoS Biol..

[24] Jianan Z., Li W., Zhang Y., Song W., Jiang H., Zhao J., Zhan Y., Teng W., Qiu L., Zhao X. (2021). Identification of glutathione transferase gene associated with partial resistance to Sclerotinia stem rot of soybean using genome-wide association and linkage mapping. Theor. Appl. Genet..

[25] Zhao X., Han Y., Li Y., Liu D., Sun M., Zhao Y., Lv C., Li D., Yang Z., Huang L. (2015). Loci and candidate gene identification for resistance to Sclerotinia sclerotiorum in soybean (*Glycine max* L. Merr.) via association and linkage maps. Plant J..

[26] Zhao F., Cheng W., Wang Y., Gao X., Huang D., Kong J., Antwi-Boasiako A., Zheng L., Yan W., Chang F. (2022). Identification of novel genomic regions for bacterial leaf pustule (BLP) resistance in soybean (*Glycine max* L.) via integrating linkage mapping and association analysis. Int. J. Mol. Sci..

[27] Zhao X., Bao D., Wang W., Zhang C., Jing Y., Jiang H., Qiu L., Li W., Han Y. (2020). Loci and candidate gene identification for soybean resistance to Phytophthora root rot race 1 in combination with association and linkage mapping. Mol. Breed..

[28] Varshney R.K., Pandey M.K., Bohra A., Singh V.K., Thudi M., Saxena R.K. (2019). Toward the sequence-based breeding in legumes in the post-genome sequencing era. Theor. Appl. Genet..

[29] Song Q., Hyten D.L., Jia G., Quigley C.V., Fickus E.W., Nelson R.L., Cregan P.B. (2013). Development and evaluation of SoySNP50K, a high-density genotyping array for soybean. PLoS ONE.

[30] Gupta P.K., Kulwal P.L., Jaiswal V. (2014). Association mapping in crop plants: Opportunities and challenges. Adv. Genet..

[31] Ibrahim A.K., Zhang L., Niyitanga S., Afzal M.Z., Xu Y., Zhang L., Zhang L., Qi J. (2020). Principles and approaches of association mapping in plant breeding. Trop. Plant Biol..

[32] Li M., Zhang Y.-W., Zhang Z.-C., Xiang Y., Liu M.-H., Zhou Y.-H., Zuo J.-F., Zhang H.-Q., Chen Y., Zhang Y.-M. (2022). A compressed variance component mixed model for detecting QTNs and QTN-by-environment and QTN-by-QTN interactions in genome-wide association studies. Mol. Plant.

[33] Sarkozi A. (2019). New Standards to Curb the Global Spread of Plant Pests and Diseases.

[34] Stukenbrock E., Gurr S. (2023). Address the growing urgency of fungal disease in crops. Nature.

[35] Zhang A., Li Y., Wang L., Wang J., Liu Y., Luan X., Liu S., Zhang J., Liu H., Yao D. (2023). Analysis of LncRNA43234-Associated ceRNA Network Reveals Oil Metabolism in Soybean. J. Agric. Food Chem..

[36] Liu Z., Li H., Wen Z., Fan X., Li Y., Guan R., Guo Y., Wang S., Wang D., Qiu L. (2017). Comparison of genetic diversity between Chinese and American soybean (*Glycine max* (L.)) accessions revealed by high-density SNPs. Front. Plant Sci..

[37] Chander S., Garcia-Oliveira A.L., Gedil M., Shah T., Otusanya G.O., Asiedu R., Chigeza G. (2021). Genetic diversity and population structure of soybean lines adapted to sub-Saharan Africa using single nucleotide polymorphism (SNP) markers. Agronomy.

[38] Abe J., Xu D., Suzuki Y., Kanazawa A., Shimamoto Y. (2003). Soybean germplasm pools in Asia revealed by nuclear SSRs. Theor. Appl. Genet..

[39] Li A.Q., Zhao C.Z., Wang X.J., Liu Z.J., Zhang L.F., Song G.Q., Yin J., Li C.S., Xia H., Bi Y.P. (2010). Identification of SSR markers using soybean (*Glycine max*) ESTs from globular stage embryos. Electron. J. Biotechnol..

[40] Tripathi N., Tripathi M.K., Tiwari S., Payasi D.K. (2022). Molecular breeding to overcome biotic stresses in soybean: Update. Plants.

[41] Yao D., Zhou J. (2023). Advances in CRISPR/Cas9-based research related to soybean [*Glycine max* (Linn.) Merr] molecular breeding. Front. Plant Sci..

[42] Varshney R.K., Bohra A., Yu J., Graner A., Zhang Q., Sorrells M.E. (2021). Designing future crops: Genomics-assisted breeding comes of age. Trends Plant Sci..

[43] Poland J., Rutkoski J. (2016). Advances and challenges in genomic selection for disease resistance. Annu. Rev. Phytopathol..

[44] Kim K.D. (1994). Susceptibility in Soybean to Red Crown Rot and Characteristics of Virulence in Calonectria Crotalariae.

[45] Nakajima T., Sakai S., Gomi T., Kikuchi A. (1994). Development of Methods for Assessing Resistance to Black Root Rot Caused by Calonectria Crotalariae in Soybean [Glycine max] and Screening for Resistant Germplasm.

[46] Brown J.K. (2015). Durable resistance of crops to disease: A Darwinian perspective. Annu. Rev. Phytopathol..

[47] Lukanda M.M., Dramadri I.O., Adjei E.A., Badji A., Arusei P., Gitonga H.W., Wasswa P., Edema R., Ochwo-Ssemakula M., Tukamuhabwa P. (2023). Genome-Wide Association Analysis for Resistance to Coniothyrium glycines Causing Red Leaf Blotch Disease in Soybean. Genes.

[48] Xiong H., Chen Y., Shi A. (2023). A genome-wide association study and genomic prediction for Phakopsora pachyrhizi resistance in soybean. Front. Plant Sci..

[49] Wen Z., Tan R., Yuan J., Bales C., Du W., Zhang S., Chilvers M.I., Schmidt C., Song Q., Cregan P.B. (2014). Genome-wide association mapping of quantitative resistance to sudden death syndrome in soybean. BMC Genom..

[50] Liu X., Huang M., Fan B., Buckler E.S., Zhang Z. (2016). Iterative usage of fixed and random effect models for powerful and efficient genome-wide association studies. PLoS Genet..

[51] Jiang G.-L., Rajcan I., Zhang Y.-M., Han T., Mian R. (2023). Soybean molecular breeding and genetics. Front. Plant Sci..

[52] Chandra S., Choudhary M., Bagaria P.K., Nataraj V., Kumawat G., Choudhary J.R., Sonah H., Gupta S., Wani S.H., Ratnaparkhe M.B. (2022). Progress and prospectus in genetics and genomics of Phytophthora root and stem rot resistance in soybean (*Glycine max* L.). Front. Genet..

[53] St (2010). Clair, D.A. Quantitative disease resistance and quantitative resistance loci in breeding. Annu. Rev. Phytopathol..

[54] Zou S., Tang Y., Xu Y., Ji J., Lu Y., Wang H., Li Q., Tang D. (2022). TuRLK1, a leucine-rich repeat receptor-like kinase, is indispensable for stripe rust resistance of YrU1 and confers broad resistance to multiple pathogens. BMC Plant Biol..

[55] Thapa G., Gunupuru L.R., Hehir J.G., Kahla A., Mullins E., Doohan F.M. (2018). A pathogen-responsive leucine rich receptor like kinase contributes to Fusarium resistance in cereals. Front. Plant Sci..

[56] Hu Y., Gong H., Lu Z., Zhang P., Zheng S., Wang J., Tian B., Fang A., Yang Y., Bi C. (2023). Variable Tandem Glycine-Rich Repeats Contribute to Cell Death-Inducing Activity of a Glycosylphosphatidylinositol-Anchored Cell Wall Protein That Is Associated with the Pathogenicity of Sclerotinia sclerotiorum. Microbiol. Spectr..

[57] Martin-Hernandez A., Dufresne M., Hugouvieux V., Melton R., Osbourn A. (2000). Effects of targeted replacement of the tomatinase gene on the interaction of Septoria lycopersici with tomato plants. Mol. Plant-Microbe Interact..

[58] Pan S., Tang L., Pan X., Qi L., Yang J. (2021). A member of the glycoside hydrolase family 76 is involved in growth, conidiation, and virulence in rice blast fungus. Physiol. Mol. Plant Pathol..

[59] Sugano S., Maeda S., Hayashi N., Kajiwara H., Inoue H., Jiang C.J., Takatsuji H., Mori M. (2018). Tyrosine phosphorylation of a receptor-like cytoplasmic kinase, BSR1, plays a crucial role in resistance to multiple pathogens in rice. Plant J..

[60] Guzha A., McGee R., Scholz P., Hartken D., Lüdke D., Bauer K., Wenig M., Zienkiewicz K., Herrfurth C., Feussner I. (2022). Cell wall-localized BETA-XYLOSIDASE4 contributes to immunity of Arabidopsis against Botrytis cinerea. Plant Physiol..

[61] Bauer K., Nayem S., Vlot A.C. (2023). β-D-XYLOSIDASE 4 modulates systemic immune signaling in Arabidopsis thaliana. Front. Plant Sci..

[62] Win K.T., Jiang C.-J. (2021). A fresh weight-based method for evaluating soybean resistance to red crown rot. Breed. Sci..

[63] Kobayashi M., Win K.T., Jiang C.-J. (2022). Soybean hypocotyls prevent Calonectria ilicicola invasion by multi-layered defenses. Front. Plant Sci..

[64] Win K.T., Maeda S., Kobayashi M., Jiang C.-J. (2021). Silicon enhances resistance to red crown rot caused by Calonectria ilicicola in soybean. Agronomy.

[65] Nishi K., Takahashi H. (1990). Influence of low temperature preservation on survival of Calonectria crotalariae. Proc. Kanto-Tosan Plant Prot. Soc..

[66] Wei T., Simko V. (2017). R Package “corrplot”: Visualization of a Correlation Matrix (Version 0.84). https://github.com/taiyun/corrplot.

[67] Purcell S., Neale B., Todd-Brown K., Thomas L., Ferreira M.A., Bender D., Maller J., Sklar P., De Bakker P.I., Daly M.J. (2007). PLINK: A tool set for whole-genome association and population-based linkage analyses. Am. J. Hum. Genet..

[68] Adamack A.T., Gruber B. (2014). PopGenReport: Simplifying basic population genetic analyses in R. Methods Ecol. Evol..

[69] Sui M., Jing Y., Li H., Zhan Y., Luo J., Teng W., Qiu L., Zheng H., Li W., Zhao X. (2020). Identification of loci and candidate genes analyses for tocopherol concentration of soybean seed. Front. Plant Sci..

[70] Zhang Y.-W., Tamba C.L., Wen Y.-J., Li P., Ren W.-L., Ni Y.-L., Gao J., Zhang Y.-M. (2020). mrMLM v4. 0.2: An R platform for multi-locus genome-wide association studies. Genom. Proteom. Bioinform..

[71] Barrett J.C., Fry B., Maller J., Daly M.J. (2005). Haploview: Analysis and visualization of LD and haplotype maps. Bioinformatics.

[72] Lyu X., Cheng Q., Qin C., Li Y., Xu X., Ji R., Mu R., Li H., Zhao T., Liu J. (2021). GmCRY1s modulate gibberellin metabolism to regulate soybean shade avoidance in response to reduced blue light. Mol. Plant.

[73] Livak K.J., Schmittgen T.D. (2001). Analysis of relative gene expression data using real-time quantitative PCR and the 2^−ΔΔCT^ method. Methods.

