# Supplementary material for: Identification and Genetic Dissection of Resistance to Red Crown Rot Disease in a Diverse Soybean Germplasm Population
Source: Plants (Basel). 2024 Mar 24;13(7):940. doi: 10.3390/plants13070940 (PMC11013609; doi:10.3390/plants13070940)
Supplement: Supplementary file 1 [file plants-13-00940-s001.zip › Table S1, S3-S4.pdf]

Table S1. Analysis of variance of three traits for RCR evaluation of the 299 worldwide soybean accessions

Trait	Variation source	*DF*	*SS*	*MS*	*F*	*Pr.*
Emergence	Replication	2	54.40	27.20	1.08	
rate	Materials	298	130948.27	439.42	17.45	<.001
	Residual	596	15012.26	25.19		
Survival	Replication	2	119.74	59.87	3.97	
rate	Materials	298	199159.96	668.32	44.33	<.001
	Residual	596	8984.59	15.07		
Disease	Replication	2	0.062	0.031	0.57	
score	Materials	298	649.35	2.18	39.83	<.001
	Residual	596	32.60	0.055		

DF= degree of freedom. *SS* = sum of squares. *MS* = mean square. *F*= *F* statistic value. *Pr.* = probability.

Table S3. Single-nucleotide polymorphism (SNP) density among the 299 worldwide soybean accessions used in this study

Chr.	Length (bp)	Length (kb)	Length (Mb)	No. of SNPs	Kbs/SNP	SNPs/Mb
1	56830220	56830.22	56.83	1552	36.6	27.31
2	48567990	48567.99	48.57	2206	22.0	45.42
3	45712413	45712.41	45.71	1545	29.6	33.80
4	52360037	52360.04	52.36	1823	28.7	34.82
5	42194057	42194.06	42.19	1828	23.1	43.32
6	51316639	51316.64	51.32	1770	29.0	34.49
7	44608799	44608.80	44.61	1922	23.2	43.09
8	47796376	47796.38	47.80	2317	20.6	48.48
9	50149215	50149.22	50.15	1675	29.9	33.40
10	51546040	51546.04	51.55	1954	26.4	37.91
11	34718252	34718.25	34.72	1470	23.6	42.34
12	40077424	40077.42	40.08	1554	25.8	38.77
13	45810724	45810.72	45.81	2327	19.7	50.80
14	49022524	49022.52	49.02	1763	27.8	35.96
15	51670112	51670.11	51.67	2245	23.0	43.45
16	37879369	37879.37	37.88	1575	24.1	41.58
17	41616549	41616.55	41.62	1833	22.7	44.04
18	57968596	57968.60	57.97	2899	20.0	50.01
19	50730824	50730.82	50.73	2120	23.9	41.79
20	47895551	47895.55	47.90	1498	32.0	31.28
	**948471711**	**948471.71**	**948.47**	**37876**	**511.74**	**802.05**

bp= base pair. Kb = kilobase. Mb= megabase. SNP = single nucleotide polymorphism.

Table S4. List of primers used for the qPCR assay

Gene ID (Wm82.a2.v1)	Primer Sequences (5'-3')	Use
Glyma.08G074600	Fw: CGATCAATATCTCCTGTCGGAA<br>Rv: CTTTCTAGGACCACCACTGTAG	qPCR assay
Glyma.08G074700	Fw: AGTATAGTTGACGAACTTGGCA<br>Rv: CTGAATGCTGAAGATTTGACGG	qPCR assay
Glyma.12G043200	Fw: GAAGCAGTTGATCGTACACATC<br>Rv: GTTCGGTGTATGGATTGTAAGC	qPCR assay
Glyma.12G043400	Fw: ATTATAACAGCGTCTACCCAGG<br>Rv: GTTTTAACAAGCAAGCCAACAC	qPCR assay
Glyma.12G043500	Fw: CAAATACTTCTCCGCCCTCC<br>Rv: TAGAGAAGCTCCAGGACGAT	qPCR assay
Glyma.12G043600	Fw: GAGGAAATGGCATTCTCGTAAC<br>Rv: CTCTGTCATTGCTGTGTTTGAA	qPCR assay
GmActin11	GGTGGTTCTATCTTGGCATC<br>CTTTCGCTTCAATAACCCTA	Reference gene
